# Spatial transcriptomic analysis reveals local effects of intratumoral fusobacterial infection on DNA damage and immune signaling in rectal cancer

**DOI:** 10.1080/19490976.2024.2350149

**Published:** 2024-05-06

**Authors:** William P. Duggan, Batuhan Kisakol, Ina Woods, Mohammedreza Azimi, Heiko Dussmann, Joanna Fay, Tony O’Grady, Barry Maguire, Ian S. Reynolds, Manuela Salvucci, Daniel J. Slade, Deborah A. McNamara, John P. Burke, Jochen H.M. Prehn

**Affiliations:** a Department of Colorectal Surgery, Beaumont Hospital, Dublin 9, Ireland; b Department of Physiology and Medical Physics, Royal College of Surgeons in Ireland, Dublin, Ireland; c RCSI Centre for Systems Medicine, Royal College of Surgeons in Ireland, Dublin, Ireland; d Department of Pathology, Beaumont Hospital, Dublin 9, Ireland; e Department of Biochemistry, Virginia Polytechnic Institute and State University, Blacksburg, VA, USA

**Keywords:** Rectal cancer, digital spatial profiling, immunogenicity, microsatellite stability, mucinous cancer, chemoresistance

## Abstract

Mucinous colorectal cancer (CRC) is a common histological subtype of colorectal adenocarcinoma, associated with a poor response to chemoradiotherapy. The commensal facultative anaerobes fusobacteria, have been associated with poor prognosis specifically in mesenchymal CRC. Interestingly, fusobacterial infection is especially prevalent in mucinous CRC. The objective of this study was therefore to increase our understanding of beneficial and detrimental effects of fusobacterial infection, by contrasting host cell signaling and immune responses in areas of high vs. low infection, using mucinous rectal cancer as a clinically relevant example. We employed spatial transcriptomic profiling of 106 regions of interest from 8 mucinous rectal cancer samples to study gene expression in the epithelial and immune segments across regions of high versus low fusobacterial infection. Fusobacteria high regions were associated with increased oxidative stress, DNA damage, and P53 signaling. Meanwhile regions of low fusobacterial prevalence were characterized by elevated JAK-STAT, Il-17, Il-1, chemokine and TNF signaling. Immune masks within fusobacterial high regions were characterized by elevated proportions of cytotoxic (CD8+) T cells (*p* = 0.037), natural killer (NK) cells (*p* < 0.001), B-cells (*p* < 0.001), and gamma delta T cells (*p* = 0.003). Meanwhile, fusobacteria low regions were associated with significantly greater M2 macrophage (*p* < 0.001), fibroblast (*p* < 0.001), pericyte (*p* = 0.002), and endothelial (*p* < 0.001) counts.

## Introduction

Fusobacteria are a genus of gram-negative anaerobes, commonly associated with gastrointestinal pathologies including inflammatory bowel disease and colorectal cancer (CRC).^1,2^ The species of fusobacteria most commonly associated with pathogenesis in CRC is *Fusobacterium nucleatum (F. nucleatum)*.^3^ In previous work, our group amongst others determined that increased *F.nucleatum* tumoral burden was associated with more advanced disease stage and worse survival in CRC.^2,4,5^ Further studies looked to explore mechanisms by which *F.nucleatum* may mediate disease pathogenesis in CRC. Findings suggested *F.nucleatum* may influence T cell and macrophage signaling, thus promoting tumor metastasis and progression.^3,6,7^ However, not all studies were able to determine a clear association between increased *F.nucleatum* tumor burden and worse outcome.^4,8–10^ Premised on the idea that the impact of fusobacteria may differ according to underlying host tumor biology, our group undertook a further study and found correlation between increased fusobacterial abundance and poor prognosis in consensus molecular subtype (CMS) IV/mesenchymal type tumors only, whilst increased fusobacterial abundance was not associated with poor outcomes in other CMS subtypes.^11^ Fusobacteria are known to be more prevalent in microsatellite instability – high (MSI-high)/CMS1 tumors, and this association may be a cofounding factor as to why some tumors with increased fusobacterial tumoral burden do not show a worse prognosis.^12,13^

Up to 15% of all CRCs are of the mucinous subtype. Mucinous CRC is a molecularly distinct subtype of colorectal adenocarcinoma known to demonstrate resistance to adjunctive chemo- and radiotherapy.^14,15^ A previous whole genome sequencing study, undertaken by our group to explore potential pathways of chemoresistance in mucinous rectal cancer, incidentally found fusobacteria to be highly abundant within mucinous tumor tissue compared to adjacent normal mucosa.^16^ We further explored this relationship in a larger mucinous cohort, and found increased fusobacterial tumoral abundance to be associated with improved outcomes in mucinous CRC.^17^ Importantly *F.nucleatum* was found to be the dominant species of fusobacteria present in mucinous colorectal cancer across both aforementioned studies.^16,17^ M2 macrophage expression is known to be greater in mucinous as compared to non-mucinous CRC.^18^ Interestingly we found increased fusobacterial tumoral load to correlate with a significant reduction in M2 macrophage expression.^17^ This relationship may partially explain the improved outcomes observed within this cohort. We also noted in this study, fusobacterial prevalence did not appear to differ significantly according to MSI status in mucinous tumors, which suggests the improved outcomes may be due to molecular interactions beyond MSI status.^17^

Previous studies on the impact of fusobacterial infection on tumor responses were largely derived from bulk sequencing or proteomics approaches. Single-cell sequencing approaches using dispersed cells have a number of critical limitations including a lack of information on spatial context. Spatial gene expression profiling allows the resolving of biological processes that accompany morphological changes in tissue across distinct regions.^19,20^ In our previous study, we optimized the use of a novel pan-*fusobacterium* antisera in formalin fixed paraffin embedded (FFPE) CRC samples.^17^ We concluded that by identifying fusobacterial infection within a digital spatial profiling platform we could decipher spatially, the precise impact of fusobacterial infection on the immune tumor microenvironment and further explore potential immunogenic and pathogenic pathways at play. In the present study, we chose selected regions of interest (ROIs) from areas of mucinous rectal tumors strongly infected with fusobacteria, and compared the transcriptome to regions of high vs. low or no fusobacterial prevalence in the same patient, and segmented the immune, and epithelial cells within each region of interest. We decided to only include MSS mucinous rectal cancer specimens to confirm the positive association between fusobacterial prevalence and mucinous CRC is a result of molecular characteristics beyond MSI. We also conducted a sub-group analysis evaluating those tumors included in our cohort who had been treated with neoadjuvant chemoradiation in advance of resection, to explore potential pathways of chemoresistance in areas of increased fusobacterial prevalence.

## Methods

### Patient samples

Formalin-fixed, paraffin-embedded (FFPE) tissue from primary tumor sections, were obtained from patients (*n* = 8) with stage II-III mucinous rectal cancer following tumor resection at the Beaumont RCSI Cancer Centre (BRCC) (See Table 1). Tissue was provided by the Beaumont Hospital Colorectal Cancer Biobank with written consent provided by all patients. Institutional ethical approval was granted by the Beaumont Research and Ethics Committee (Reference 21/98). Mucinous tumors were defined by a consultant histopathologist as those with greater than 50% of the tumor composed of mucin.^21^ The Mandard tumor regression grading score was used to assess response to neoadjuvant chemoradiotherapy.^22^ Microsatellite instability (MSI) screening status was assessed by immunohistochemistry and other clinical data was extracted from a prospectively maintained patient database.

**Table 1.** Clinical and pathological characteristics of the included patient cohort.

	Age	Sex	pT Stage	pN Stage	AJCC	MSI Status	Neoadjuvant Treatment	Grade (Differentiation)	TRG
Case A	30–35	M	3	2	III	MSS	Yes	Moderate	TRG5
Case B	80–85	M	3	0	II	MSS	Yes	Moderate	TRG4
Case C	70–75	F	2	1	III	MSS	No	Moderate	NA
Case D	75–80	M	3	1	III	MSS	Yes	Poor	TRG4
Case E	70–75	F	3	0	II	MSS	Yes	Moderate	TRG4
Case F	60–65	M	4	2	III	MSS	No	Moderate	NA
Case G	70–75	M	3	0	II	MSS	Yes	Moderate	TRG3
Case H	75–80	M	1	1	III	MSS	No	Moderate	NA

### Sample preparation

To prepare for digital spatial profiling (DSP), 5 µm thick full-face FFPE sections were cut, baked and mounted on individual slides. Samples underwent standard de-paraffinisation which involved; 3 × 5 min washes with xylene, followed by 2 × 5 min washes in 100% ethanol, followed by a 1 × 5 min wash in 95% ethanol. Next, antigen target retrieval was performed, samples were placed in 1X Tris EDTA (PH 9.0) (Abcam) at 100°C for 20 min, next samples were placed in 1 µg/mL proteinase K at 37°C for 15 min. Samples were then fixed in 10% neutral buffered formalin (NBF) for 5 min, this was followed by 2 × washes in NBF stop buffer.

Prepared slides were incubated with immunofluorescent antibodies and GeoMx Cancer Transcriptome Atlas (CTA, V.2.0) profiling reagents simultaneously. An overnight in situ hybridization was performed with CTA RNA probes. Slides were washed twice at 37°C for 25 min with 50% formamide/2X SSC buffer to remove unbound probes. Slides were next blocked with Buffer W (Nanostring) for 30 min at 37°C. This was followed by the addition of 1:300 dilution of the fusobacterial antisera for 1 h at 37°C. The pan-fusobacterium outer membrane antisera was produced and characterized as previously described.^23^ Creation of this antisera involved purification of antibodies produced following injection of rabbits with purified total membrane proteins from 11 strains of fusobacteria that span seven species (*F. nucleatum subsp. nucleatum* 23726, *F. nucleatum subsp. animalis* 7_1, *F. nucleatum* subsp. polymorphum 10,953, *F. nucleatum subsp. vincentii* 49256, *F. periodonticum* 2_1_31, *F. varium* 27725, *F. ulcerans* 49185, *F. mortiferum* 9817, *F. gonidiaformans* 25563, *F. necrophorum subsp. necrophorum* 25286, and *F. necrophorum subsp. funduliforme* 1_1_36S). The antisera was optimized for use with FFPE tissue, as previously described.^17^ To verify the validity of the antisera, sections from 8 tumors which had previously undergone whole genome sequencing, were stained with the antisera. Fusobacterial burden was quantified and levels were compared with fusobacterial relative abundance as interpreted previously from existing sequencing data.^16,17^ Slides were next washed twice for 2 min in 2 × SSC wash buffer and incubated for 1 h with STAR 635 goat anti-rabbit antibody (Sigma-Aldrich) diluted in buffer W (Nanostring). After 3 further washes with 2 × SSC, slides were finally incubated for 1 h with Syto 13 (Nuclear stain) (Nanostring), pancytokeratin conjugated to Alexa Fluor 532 (tumor epithelial cells) (Nanostring) and CD45 conjugated to 594 (immune cells) (Nanostring). Exposure time was set to 300 ms for the 594, 532 and 635 channels and 100 ms for the 488 channel. Stained slides were loaded onto the GeoMx instrument and scanned.

### Digital spatial profiling

**Figure 1.**
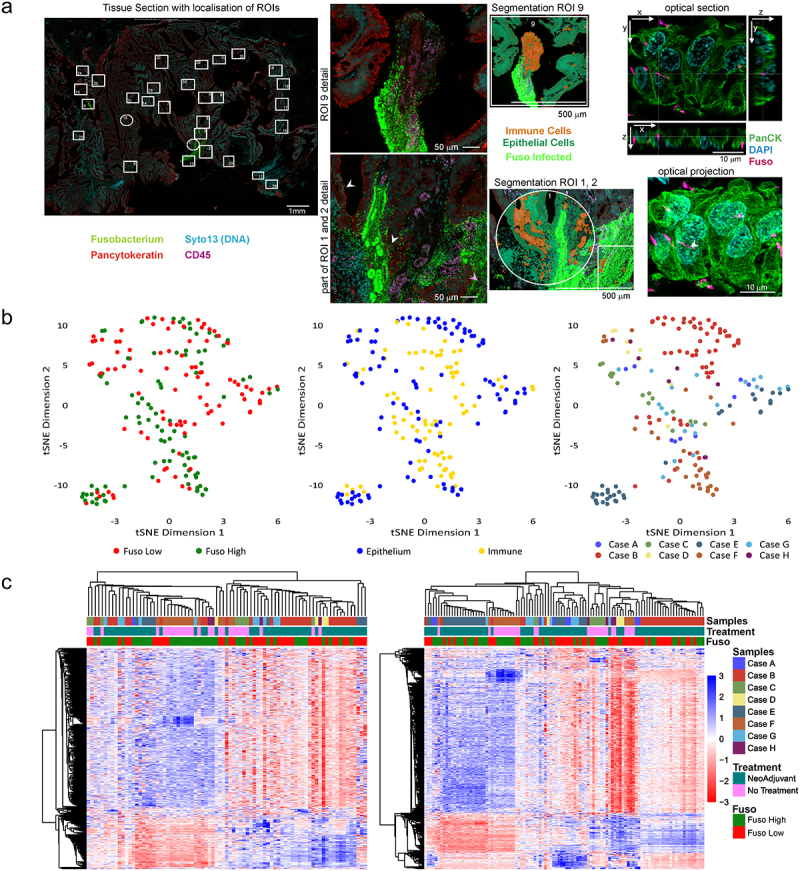
(a) Representative example of a mucinous rectal cancer sample imaged on the GeoMx platform. Pancytokeratin = green, nuclear stain (Syto13) = cyan, CD45 (immune) = magenta, and fusobacterium antisera = green. Left: overview of the regions analyzed in the tissue, middle left: higher magnification of 2 individual ROIs showing fusobacteria close to pancytokeratin (white arrowhead) and CD45 (magenta arrowhead) labels. Middle right: artificial overlay of tissue segmentation is indicated for this ROI, visualizing immune (orange) and epithelial (green) segments. On the right a small subset of a subsequent confocal optical sections and z-maximum intensity projection shows fusobacterial (magenta) inside a pancytokeratin (green) labeled cell close to nucleus (cyan, crosshair and white arrowhead). (b) Dimensionality reduction visualization of all AOIs according to overall gene expression profiles by tSNE. The tSNE plots are annotated by fusobacteria high/Low (left), histological region (middle) and sample ID (right). (c) Heatmaps of gene expression (n = 1825) using unsupervised clustering for epithelial AOIs (right) and immune AOIs (left). Heatmaps are annotated by histological region and sample ID. AOI, areas of illumination; RC, rectal cancer; ROI, region of interest; tSNE, *t*-distributed stochastic neighbour embedding.

For each tumor, geometrical regions of interest (ROIs) were selected to include an equal number of regions of high and low fusobacterial infection (Figure 1(a)). Separate sections from the selected specimens were pre-stained with the aforementioned morphology markers and the fusobacterial antisera to confirm the presence and location of fusobacterial foci within each tumor specimen. Analogous tumor locations were selected with the assistance of a trained pathologist, to include similar areas of tumor with no apparent positive fusobacterial staining. Total fusobacterial staining scores from each ROI were calculated. Fusobacterial scores from fusobacteria ‘high’ ROIs ranged from 2.447–30.550. Whereas many of the fusobacteria ‘low’ regions were found to have no fusobacteria staining, some were found to contain low fusobacterial scores. Fusobacterial scores from fusobacteria ‘low’ ROIs ranged from 0–0.004. We used geometrical shapes, including rectangular shapes, circles and polygons, to specifically select tumor regions. The resulting surface areas and number of nuclei for each ROI varied depending on the respective size of the distinct regions and the cellular density but each ROI must fit into one field of view which is 665 × 665 µm^2^ (Supplemental Table 1). The number of ROIs per tumor also varied significantly according to the prevalence of fusobacteria in each tumor. ROIs were segmented into pan-cytokeratin positive epithelial cells and CD45-positive immune cells. The segmentation process utilized a random forest classifier to categorize pixels based on local variables at several scales, including color, texture, and edge-ness. A sharpness adjustment method was employed to improve segmentation accuracy (see data availability statement for details). Each ROI was UV-illuminated twice, once for the epithelial segment and once for the immune segment. Each individual segment is referred to as an area of interest (AOI). Exposure to the UV light released the indexing oligonucleotides which were collected with a microcapillary and deposited in a 96-well plate for subsequent processing. Probes were collected from 184 areas of interest across the 8 tumor samples.

### Library preparation and sequencing

Sequencing libraries were generated by PCR from the photo-released indexing oligos and AOI-specific Illumina adapter sequences. Unique i5 and i7 sample indices were added, each PCR reaction used 4 μl of indexing oligonucleotides, 4 μl of indexing PCR primers and 2 μl of Nanostring 5X PCR Master Mix. Thermocycling conditions were 37°C for 30 min, 50°C for 10 min, 95°C for 3 min; 18 cycles of 95°C for 15 s, 65°C for 1 min, 68°C for 30 s; and 68°C for 5 min. PCR reactions were pooled and purified twice using AMPure XP beads (Beckman Coulter, A63881), according to the manufacturer’s protocol. Samples were bioanalyzed as a means of quality control (QC) to out rule potential contamination. Pooled libraries were single-sequenced at 27 base pairs and with the single-index workflow on an Illumina NovaSeq SP instrument (Genomics Core, KU-Leuven, Belgium).

### DSP data processing and normalisation

FastQ files were converted into DCC files according to the manufacturer’s pipeline. The “GeomxTools” package in R, was used for QC, filtering and processing of the samples.^24^ Filtering steps included selecting default probe and segmenting QC flags from the “GeomxTools” library. The study was designed with “GeoMx Cancer Transcriptome Atlas” and expression profiles of 1812 genes were calculated using this panel. After the QC, we aggregated probe counts to target gene levels and obtained the gene expression counts. After aggregating probe counts to the gene count, quantile normalization was performed. For the downstream analysis, we used the “Seurat” package in R.^25^

### Immunofluorescence immunohistochemistry

To determine the spatial localization of fusobacteria within or in proximity to tumor cells, we performed staining of sections subsequent to sections analyzed by Nanostrong. 5 µm thick full-face FFPE sections were cut, baked and mounted on individual slides. Samples underwent standard deparaffinization as previously described above. Prepared slides were blocked with Buffer W (Nanostring) for 30 min at 37°C. Sections were stained using the fusobacterium antisera described above as well as pancytokeratin conjugated to Alexa 488 (eBioscience, Thermo Fisher) and DAPI (Merck) as a DNA marker. We then performed confocal z-stacks of areas showing fusobacteria using an LSM 980 Airyscan 2 (Carl Zeiss) in 4Y mode with 40 × 1.3 NA Objective resulting in a resolution of Δ x, Δ y = 140 nm and Δz = 450 nm to allow for more precise localization of fusobacteria.

We also performed immunofluorescence immunohistochemistry on subsequent sections of rectal cancer specimens to validate our transcriptomic findings at a protein level. This was followed by the addition of 1:100 dilution of RAD21 antibody (Cell Signalling) for 1 h at 37°C. RAD21 is a protein responsible for post-replicative DNA repair, and the prevention of inappropriate recombination between repetitive regions.^26^ Slides were next washed twice for 2 min in 2 × SSC wash buffer and incubated for 1 h with STAR 635 goat anti-rabbit antibody (Sigma-Aldrich) diluted in buffer W (Nanostring). After 3 further washes with 2 × SSC, slides were finally incubated for 1 h with Syto 13 (Nuclear stain) (Nanostring), pancytokeratin conjugated to Alexa Fluor 532 (Nanostring) and CD45 conjugated to 594 (Nanostring). Exposure time was set to 300 ms for the 532, 594 and 635 channels and 100 ms for the 488 channel. Stained slides were loaded onto the GeoMx instrument and scanned. Images were processed using Imagej (BigDataViewer) to establish whether RAD21 expression coincided with regions of increased fusobacterial prevalence.

### Bioinformatics and statistical analyses

After coercing the GeoMx objects into Seurat objects, we performed several statistical analyses. We identified differentially expressed genes between fusobacteria low and fusobacteria high regions in the epithelium and immune masks. We also identified differentially expressed genes between samples that received neoadjuvant therapy and those that were treatment naïve. We investigated the pathway enrichment levels between different groups and regions of interest. Gene set enrichment analysis (GSEA) was done on normalized counts by using the “fgsea” library in R.^27^ The normalized enrichment scores (NESs) were calculated using the Wilcoxon rank sum test. Genes were sorted according to fold changes from the differential expressions analyses results. Then gene set enrichment was performed based on the ranked gene lists. A high normalized enrichment score (NES) indicates an overrepresentation of the genes in the fusobacteria high ROIs, and a low NES indicates an overrepresentation of the genes in the fusobacteria low samples. Volcano plot of differentially expressed genes between fusobacteria high and low regions were analyzed by Wilcoxon rank sum test. Gene lists were selected from the NanoString Cancer Transcriptome Atlas RNA Probe list. Immune cell signatures for each sample were calculated using CIBERSORT and MCPcounter packages using all AOIs.^28,29^

SpatialDecon described by Danaher *et al*. was used to quantify signatures of pericytes, smooth muscle cells, plasma cells, endothelial cells, and fibroblasts present in the tumor mask of the spatial transcriptomics datasets.^30^ Scores for each cell type were generated with the ‘SpatialDecon’ (v1.12) package in R (v4.3) and figures were generated using Python (v3.9) and R.

### Validation of ‘fusobacterium high’ and ‘fusobacterium low’ gene signatures

We performed a search of the cancer genome atlas dataset (TCGA) to identify cases of CRC eligible for inclusion. Institutional approval was not required for these open-access data. The inclusion criteria specified stages 1 to 4; mucinous and non-mucinous colorectal adenocarcinoma. Patients of both genders and all age groups and ethnicities were eligible for inclusion in the analysis. Data were collated and harmonized from the GDC Legacy Archive and the TCGA-Clinical Data Resource publication.^31^ We restricted the analysis to patients of the TCGA-Colorectal Adenocarcinoma (COAD)-Rectal Adenocarcinoma (READ) cohort that have both clinical information and fusobacteria data [*n* = 594 of 631 candidate cases (94%)]. Fusobacterial levels of the TCGA datasets were previously calculated by Salvucci et al..^11^ The 75th percentile of the Fusobacteriales load was used as a cutoff and the upper (75th) percentile indicated the “Fuso High” and the lower percentiles marked the “Fuso Low”.

## Results

We selected ROIs from areas of tumor containing fusobacteria and compared the transcriptome to regions of low or no fusobacterial prevalence. We included 53 fusobacteria high ROIs and 53 fusobacteria low ROIs across 8 MSS rectal cancer specimens (see supplemental Table 1.). We segmented the immune and epithelial cells within each region of interest in order to establish differential immune and epithelial cell signaling according to the prevalence of fusobacteria within specific ROIs. Data exploration using dimension reduction by t Distribution Stochastic Neighbour Embedding (tSNE) revealed high and low fusobacterial AOIs were separated within both epithelium and immune segments. We also observed that AOIs corresponding to high and low fusobacterial regions, formed small sub-clusters by sample (Figure 1(b)), most likely representing inter-patient variability regarding tumor-associated transcriptomic profiles.

We next visualized expression of all 1825 CTA genes in unsupervised clustered heatmaps, per patient sample (Figure 1(c)). In total, 95 genes were upregulated in the fusobacteria high epithelial segments and 69 genes were upregulated in the corresponding immune segments (*p* < .01). Meanwhile, 354 genes were upregulated in the fusobacteria low epithelial segments, whilst 67 genes were upregulated in the fusobacteria low immune segments (*p* < .01).

### Fusobacterial infection is associated with an anti-tumor immune response in mucinous rectal cancer

Previously, our group and others determined macrophage and T-cell recruitment may differ significantly in CRC according to the prevalence of fusobacteria.^6,7,17^ HLA-DRA, HLA-DRB, and Alpha-2-macroglobulin (A2M) were all found to be up-regulated in fusobacteria high immune segments in our analysis. HLA-DRA and HLA-DRB, are Class II HLA molecules that are constitutively expressed in antigen-presenting cells. A2M is an extracellular macromolecule mainly known for its role as a protease inhibitor, A2M lures active proteases into its molecular cage and subsequently ‘flags’ them for elimination.^32^ By contrast, pro-inflammatory and pro-migratory chemokines and cytokines such as CXCL8 and interleukin-1B (IL1B) were specifically up-regulated in fusobacteria low ROIs.

**Figure 2.**
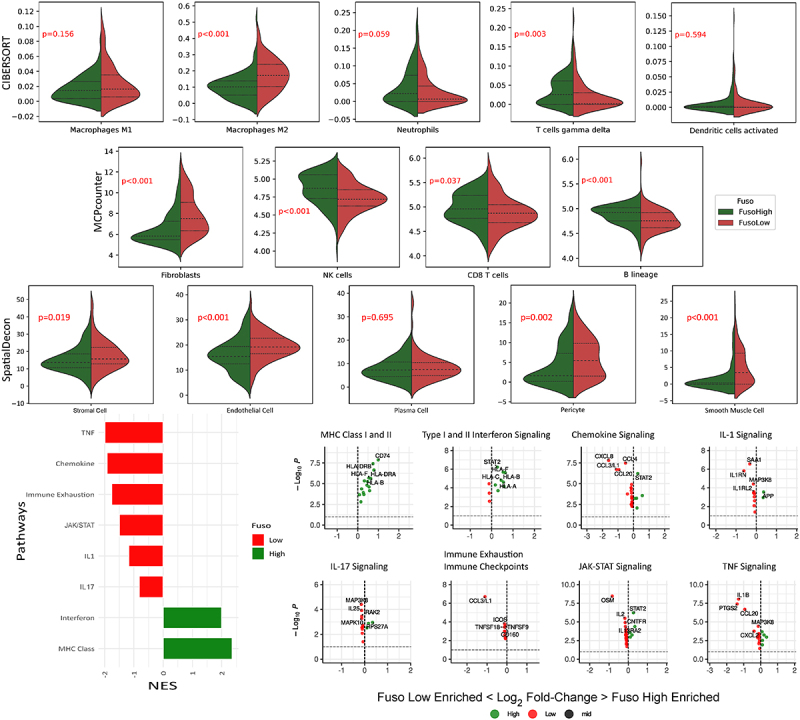
Violin plots of differentially expressed immune cells identified in the fusobacteria high versus low regions by wilcoxin rank sum test. Genes are sorted according to fold changes from the differential expressions analyses results. Gene set enrichment is performed based on the ranked gene lists. A high normalized enrichment score (NES) indicates an overrepresentation of the genes in the fusobacteria high ROIs, and a low NES indicates an overrepresentation of the genes in the fusobacteria low samples.

We used CIBERSORT and MCPcounter packages, to enrich and deconvolute immune cell expression within our selected ROI’s and compared immune cell expression in the fusobacteria high versus low regions (Figure 2). Fusobacteria high regions were associated with significantly greater expression of cytotoxic (CD8+) T cells (*p* = 0.037), natural killer (NK) cells (*p* < .001), B-cells (*p* < .001), and gamma delta T cells (*p* = 0.003). We used the Spatialdecon package to explore different cell types in the tumor cell mask. We identified significantly greater M2 macrophage (*p* < .001), fibroblast (*p* < .001), pericyte (*p* = .002) and endothelial cell (*p* < .001) infiltration within the tumor mask of fusobacterial low ROI’s (Figure 2).

We next conducted gene set enrichment analysis to establish whether the observed differences in immune cell expression corresponded with respective immune signaling pathways (Figure 2). Both MHC class I and II, and Interferon I and II signaling pathways were activated in fusobacteria high ROIs. Meanwhile fusobacteria low regions were characterized by elevated JAK-STAT, Il-17, Il-1, chemokine and TNF signaling. Interestingly, genes associated with immune exhaustion were also up-regulated in fusobacteria low ROIs.

### Fusobacterial prevalence is associated with increased oxidative stress, DNA damage and impaired lipid metabolism in tumor cells

**Figure 3.**
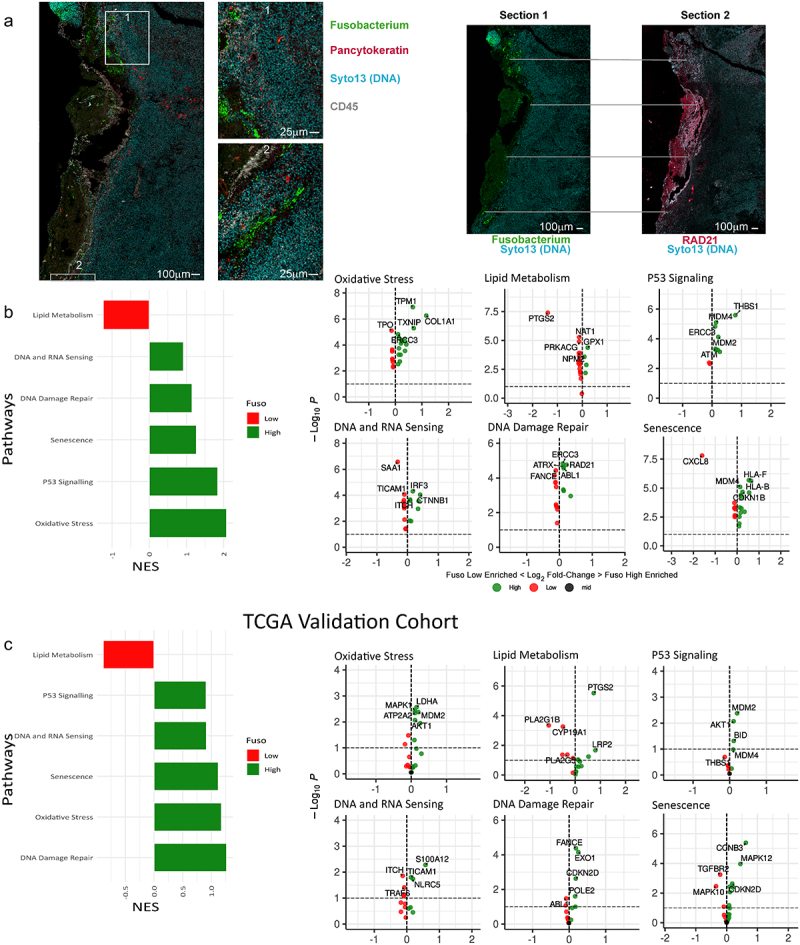
(a) Imaging of mucinous sample with GeoMx. Left: image of a tissue section showing fuso bacteria with insets (middle left) showing the localization of fusobacteria in higher magnification. On the right fusobacterial staining is shown together with RAD21 staining of a subsequent section of the same tissue highlighting examples of the close localization of fusobacterial and RAD21 staining with gray lines. (b) GSEA showing DNA damage and repair associated signaling pathways, that are differentially regulated in high versus low fusobacterial regions. A high normalized enrichment score (NES) indicates an overrepresentation of the genes in the fusobacterial high ROIs, and a low NES indicates an overrepresentation of the genes in the fusobacteria low samples. (c) TCGA validation cohort, which includes 594 patients with CRC (non-mucinous; *n* = 522, mucinous; *n* = 72). The 75th percentile of the fusobacteria load is used as a cutoff and the upper (75th) percentile indicates the “fuso high” and the lower percentiles mark the “fuso low”.

We next investigated differences in the gene expression profiles of tumor cells in fusobacteria high and low ROIs in more detail. Genes associated with DNA damage and repair signaling, including; RPS27A, THBS1, and CDKN1B were all found to be upregulated in regions of high fusobacterial prevalence compared to low fusobacterial ROIs (Figure 3(b)). Other genes upregulated in high fusobacteria regions included the tumor suppressor TPM1, the collagen family gene member COL1A1, and TXNIP, an inhibitor of cell proliferation. PTGS2 was up-regulated in fusobacteria low ROIs. Gene set enrichment analysis revealed fusobacteria high regions were associated with DNA damage and repair signaling pathways, P53 signaling, DNA and RNA sensing and senescence signaling pathways (Figure 3(b)). Whilst fusobacteria low regions were associated with enhanced lipid metabolism (Figure 3(b)). We validated these findings using the TCGA dataset, which included 594 patients with CRC. To increase sample size, validation included data from mucinous and non-mucinous tumors. Interestingly fusobacteria high tumors in the TCGA dataset were also associated with DNA damage and repair signaling pathways, P53 signaling, DNA and RNA sensing and senescence signaling pathways (Figure 3(c)). Fusobacteria high regions were also associated with increased DNA damage on a protein level as detected by staining of tumor sections with an antibody detecting RAD21, a DNA double-strand break repair protein. We found strong correlation between regions of enhanced RAD21 and high fusobacterial levels (Figure 3(a)).

### Fusobacterial high regions are associated with increased autophagy and cell differentiation signaling in response to neoadjuvant chemoradiotherapy

**Figure 4.**
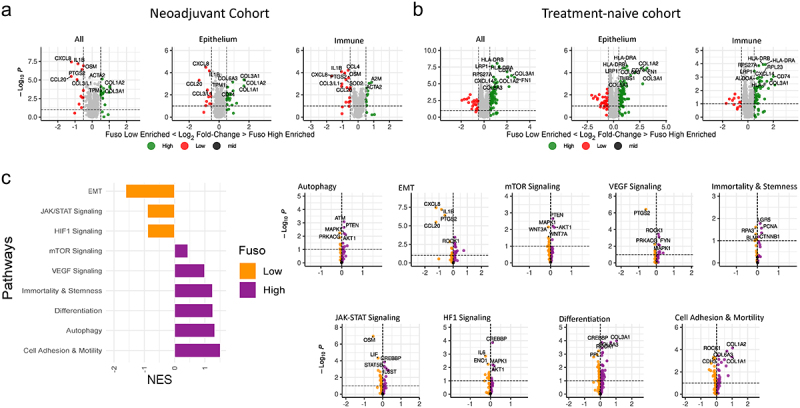
(a) Volcano plot of differentially expressed genes between patients who did or did not receive neoadjuvant chemoradiotherapy in advance of surgical resection. (b) Volcano plot of differentially expressed genes between fusobacteria high and low regions from patients who were in receipt of neoadjuvant treatment. (c) Volcano plot of differentially expressed genes between fusobacteria high and low regions of treatment naïve patients who were not in receipt of neoadjuvant treatment. (d) GSEA analysis showing signaling pathways associated with chemoresistance that are differentially regulated in high versus low fusobacteria regions. A high normalized enrichment score (NES) indicates an overrepresentation of the genes in the fusobacteria high ROIs, and a low NES indicates an overrepresentation of the genes in the fusobacteria low samples.

Mucinous CRC demonstrates resistance to numerous adjunctive systemic therapies.^14,33^ *F. nucleatum* has previously been found to promote chemoresistance in CRC by modulating autophagy.^34^ To further explore whether a relationship exists between fusobacterial prevalence and chemoresistance, in mucinous rectal cancer, we conducted a subgroup analysis comparing gene expression profiles between fusobacterial low and high regions in patients who received neoadjuvant chemoradiation (Figure 4(a, b)), and patients who were treatment naïve in advance of surgical resection (Figure 4(c)). Gene set enrichment analysis on patients who were in receipt of neoadjuvant therapy showed that autophagy, differentiation, immortality, and stemness signaling pathways were activated in high fusobacteria regions (Figure 4(d)). VEGF and cell adhesion and motility signaling pathways were also activated in high fusobacterial ROIs (Figure 4(d)).

## Discussion

The primary objective of this study was to develop an understanding of how fusobacteria positively or negatively impacts on immune and tumor cell signaling in colorectal cancer, using mucinous MSS rectal cancer as a clinically highly relevant tumor. Our analysis suggests fusobacteria are associated with a strong anti-tumor immune response and that this may occur as a consequence of local DNA damage and oxidative stress. Fusobacteria high regions were characterized by elevated proportions of strongly anti-tumoral immune cells including cytotoxic (CD8+) T cells, and gamma delta T cells, whilst fusobacteria low regions were associated with enhanced M2 macrophage, pericyte, endothelial cell and fibroblast expression. Our secondary objective was to explore further, the potential role played by fusobacteria in promoting chemoresistance in mucinous rectal adenocarcinoma. Sub-group analysis of patients in receipt of neoadjuvant chemoradiation, suggests fusobacteria may promote autophagy, differentiation, immortality, and stemness, in mucinous rectal cancer, all of which are pathways known to be associated with chemoresistance.

In this current study, we found fusobacteria high regions to be associated with enhanced CD8+ (cytotoxic) T cell, NK cell and gamma delta T cell expression, whilst fusobacteria low regions were associated with increased M2 macrophage expression. CD8+ cytotoxic T cells and NK cells are the cornerstone of the anti-tumor immune response in CRC.^35^ Meanwhile gamma delta T cells can effectively recognize and kill CRC cells, thereby suppressing tumor progression.^36^ High-density M2 macrophage infiltration is associated with poor survival in solid-organ tumors.^18^ These cells have been implicated in tumor migration, invasion, and have been found to induce an attenuated anti-tumor immune response.^18^ Pericytes are thought to actively contribute to classic cancer hallmarks, and may differentiate toward stromal fibroblasts in specific cancer tumor environments to promote metastasis, and evasion of immune destruction.^37^ Collectively, this altered immune environment could potentially explain the observed improved outcomes in patients with mucinous CRC with elevated fusobacterial tumoral abundance.^17^

Existing evidence suggests fusobacteria may promote DNA damage in cancer.^38–40^ DNA damage and oxidative stress signaling pathways were found to be up-regulated in fusobacteria high ROI’s on transcriptomic analysis in this study. We also found fusobacteria to be associated with DNA damage on a protein level. Hseuh et al. previously found *F. nucleatum* to be associated with increased miR-205-5p expression, which was found to suppress MLH1, MSH2, and MSH6 expression via the TLR4- and MYD88-dependent pathways in head and neck squamous cell cancer, resulting in deficient mismatch repair, DNA damage, and cell proliferation.^38^ Guo et al. provided evidence that *F.nucleatum* may induce DNA damage and cell growth in CRC through FadA-dependent activation of the E-cadherin/β-catenin pathway, leading to up-regulation of chk2,^39^ whilst Okita et al. demonstrated evidence that *F.nucleatum* produces factors that induce γ-H2AX, a hallmark of DNA double-strand breaks, in infected CRC cells.^40^ DNA damage and failure of cells to adequately repair DNA, results in an elevated cellular mutational burden.^41^ Neoantigens are produced as a result and detection of these proteins, causes arousal of the innate and adaptive immune response.^41^ It is our assertion that this is the most likely mechanism through which a strong anti-tumor immune response is observed in regions of high fusobacterial prevalence. Interestingly, PTGS2 was up-regulated in fusobacteria low ROIs in our analysis, which is in contrast to previous studies which demonstrated enhanced expression in tumors with increased fusobacterial infection.^42,43^ This is a further example of how the molecular impact of fusobacterial infection may differ significantly according to underlying host tumor biology.

Our findings also suggest that lipid metabolism may be inhibited in regions of high fusobacterial prevalence in mucinous rectal cancer, whilst oxidative stress was found to be enhanced in the same regions. Impaired lipid metabolism is linked to alterations in the oxidant/antioxidant balance, leading to cellular lipotoxicity, lipid peroxidation, chronic endoplasmic reticulum (ER) stress, and mitochondrial dysfunction.^44,45^ Fusobacteria have previously been found to interfere with lipid metabolism.^45,46^ *F. nucleatum* have been implicated in nonalcoholic fatty liver disease progression, and are also associated with accelerated atherosclerosis through macrophage mediated promotion of aberrant lipid metabolism^45,46^ *F. nucleatum* are thought to inhibit nicotinamide adenine dinucleotide (NAD+)‘s salvage metabolism in the liver, leading to increase production of oxygen-free radicals, which result in DNA damage and inflammation.^45^ We suggest further studies to explore this avenue as a potential mechanism through which these bacteria may cause DNA damage in this context.

Mucinous CRC demonstrates resistance to numerous adjunctive systemic therapies.^14,33^ A sub-analysis of our neoadjuvant cohort identified autophagy, differentiation, immortality and stemness as signaling pathways up-regulated in regions of high fusobacterial infection. *F.nucleatum* have previously been found to promote chemoresistance in CRC by modulating autophagy.^34^ Autophagy protects cells against metabolic stress and prevents cell death and senescence. Autophagy can also influence cell fate and regulate stem cell quiescence, activation, differentiation, and self-renewal in response to chemotherapy.^47,48^ Our findings should prompt further studies into the potential role of *fusobacterium* in promoting chemoresistance in mucinous CRC.

Though our findings regarding the impact of fusobacterial infection in mucinous rectal cancer are important, there are a number of limitations to our study. Firstly, though the number of ROIs included for analysis were significant, they were derived from a relatively small number of patient samples. Nonetheless, we feel the conclusions derived here are of significant relevance to prompt further investigations into larger patient cohorts. Additionally, we were also able to validate our spatial transcriptomics-derived signatures in the TCGA cohort. The antisera utilized represents a pan-fusobacteria antisera. There are 14 known species of fusobacteria. These include *Fusobacterium*; *nucleatum, necrophorum* and *varium*.^49^ Though evidence suggests the activity of these species may differ in certain contexts, all species are associated with CRC, and evidence from our prior studies suggests *F.nucleatum* tends to be by far the dominant species in mucinous CRC.^16,17,49^ Nonetheless, further analysis of the impact of individual species in this context is warranted. An absence of information pertaining to the presence and impact of other bacterial species is a further limitation of this work. We know from recent studies that various bacterial species tend to co-populate poorly vascularized microniches within tumoral tissue.^50^ Therefor it is likely that other species may also have contributed toward the differential pathway signaling observed amongst fusobacteria high and low ROI’s in this study.

Our spatial transcriptomic profiling demonstrated the complexity of host – bacterial interactions. Fusobacterial infection promoted an immunogenic microenviroment associated with elevated oxidative stress and DNA damage. Meanwhile, in the same patients, low fusobacterial regions were characterized by the presence of immune cells associated with promoting disease progress and metastasis. Sub-group analysis of patients in receipt of neoadjuvant chemoradiation, suggests fusobacteria may promote autophagy, differentiation, immortality and stemness, signaling pathways in mucinous rectal cancer all known to be associated with chemoresistance.

## Supplementary Material

Supplemental Material

## Data Availability

The data that support the findings of this study are available on request from the corresponding author, JHMP. The data are not publicly available due to their containing information that could compromise the privacy of research participants.
