# Supplementary material for: Spatial transcriptomic analysis reveals local effects of intratumoral fusobacterial infection on DNA damage and immune signaling in rectal cancer
Source: Gut Microbes. 2024 May 6;16(1):2350149. doi: 10.1080/19490976.2024.2350149 (PMC11086019; doi:10.1080/19490976.2024.2350149)
Supplement: Supplemental Material [file KGMI_A_2350149_SM5715.docx]

| **AOI_ID** | **roi** | **Fuso** | **Cell type** | **Cell Count** |
| --- | --- | --- | --- | --- |
| DSP-1001660010710-A-A02 | 001 | 1 | Immune | 484 |
| DSP-1001660010710-A-A03 | 001 | 1 | Epi/Stroma | 2750 |
| DSP-1001660010710-A-A04 | 002 | 1 | Immune | 239 |
| DSP-1001660010710-A-A05 | 002 | 1 | Epi/Stroma | 2454 |
| DSP-1001660010710-A-A06 | 003 | 1 | Immune | 220 |
| DSP-1001660010710-A-A07 | 003 | 1 | Epi/Stroma | 1789 |
| DSP-1001660010710-A-A08 | 004 | 1 | Immune | 252 |
| DSP-1001660010710-A-A09 | 004 | 1 | Epi/Stroma | 2404 |
| DSP-1001660010710-A-A10 | 005 | 0 | Immune | 1928 |
| DSP-1001660010710-A-A11 | 005 | 0 | Epi/Stroma | 1324 |
| DSP-1001660010710-A-A12 | 006 | 0 | Immune | 386 |
| DSP-1001660010710-A-B01 | 006 | 0 | Epi/Stroma | 1947 |
| DSP-1001660010710-A-B02 | 007 | 0 | Immune | 307 |
| DSP-1001660010710-A-B03 | 007 | 0 | Epi/Stroma | 2796 |
| DSP-1001660010710-A-B04 | 008 | 0 | Immune | 268 |
| DSP-1001660010710-A-B05 | 008 | 0 | Epi/Stroma | 3956 |
| DSP-1001660010710-A-B06 | 001 | 1 | Epi/Stroma | 913 |
| DSP-1001660010710-A-B07 | 001 | 1 | Immune | 500 |
| DSP-1001660010710-A-B08 | 002 | 1 | Epi/Stroma | 347 |
| DSP-1001660010710-A-B09 | 003 | 1 | Epi/Stroma | 738 |
| DSP-1001660010710-A-B10 | 004 | 1 | Epi/Stroma | 1367 |
| DSP-1001660010710-A-B11 | 005 | 0 | Epi/Stroma | 1305 |
| DSP-1001660010710-A-B12 | 006 | 0 | Epi/Stroma | 905 |
| DSP-1001660010710-A-C01 | 007 | 0 | Epi/Stroma | 1954 |
| DSP-1001660010710-A-C02 | 008 | 0 | Epi/Stroma | 1556 |
| DSP-1001660010710-A-C03 | 001 | 1 | Immune | 354 |
| DSP-1001660010710-A-C04 | 001 | 1 | Epi/Stroma | 1565 |
| DSP-1001660010710-A-C05 | 002 | 1 | Immune | 161 |
| DSP-1001660010710-A-C06 | 002 | 1 | Epi/Stroma | 1970 |
| DSP-1001660010710-A-C07 | 003 | 1 | Immune | 577 |
| DSP-1001660010710-A-C08 | 003 | 1 | Epi/Stroma | 1849 |
| DSP-1001660010710-A-C09 | 004 | 1 | Immune | 415 |
| DSP-1001660010710-A-C10 | 004 | 1 | Epi/Stroma | 1182 |
| DSP-1001660010710-A-C11 | 005 | 1 | Immune | 576 |
| DSP-1001660010710-A-C12 | 005 | 1 | Epi/Stroma | 1864 |
| DSP-1001660010710-A-D01 | 006 | 1 | Immune | 208 |
| DSP-1001660010710-A-D02 | 006 | 1 | Epi/Stroma | 3024 |
| DSP-1001660010710-A-D03 | 007 | 1 | Immune | 332 |
| DSP-1001660010710-A-D04 | 007 | 1 | Epi/Stroma | 1485 |
| DSP-1001660010710-A-D05 | 008 | 1 | Immune | 303 |
| DSP-1001660010710-A-D06 | 008 | 1 | Epi/Stroma | 1884 |
| DSP-1001660010710-A-D07 | 009 | 1 | Immune | 128 |

| DSP-1001660010710-A-D08 | 009 | 1 | Epi/Stroma | 1547 |
| --- | --- | --- | --- | --- |
| DSP-1001660010710-A-D09 | 010 | 1 | Immune | 65 |
| DSP-1001660010710-A-D10 | 010 | 1 | Epi/Stroma | 1977 |
| DSP-1001660010710-A-D11 | 011 | 1 | Immune | 407 |
| DSP-1001660010710-A-D12 | 011 | 1 | Epi/Stroma | 1378 |
| DSP-1001660010710-A-E01 | 012 | 1 | Immune | 108 |
| DSP-1001660010710-A-E02 | 012 | 1 | Epi/Stroma | 940 |
| DSP-1001660010710-A-E05 | 014 | 1 | Immune | 735 |
| DSP-1001660010710-A-E06 | 014 | 1 | Epi/Stroma | 2066 |
| DSP-1001660010710-A-E07 | 015 | 0 | Immune | 247 |
| DSP-1001660010710-A-E08 | 015 | 0 | Epi/Stroma | 1012 |
| DSP-1001660010710-A-E09 | 016 | 0 | Immune | 188 |
| DSP-1001660010710-A-E10 | 016 | 0 | Epi/Stroma | 3613 |
| DSP-1001660010710-A-E11 | 017 | 0 | Immune | 194 |
| DSP-1001660010710-A-E12 | 017 | 0 | Epi/Stroma | 2841 |
| DSP-1001660010710-A-F01 | 018 | 0 | Immune | 145 |
| DSP-1001660010710-A-F02 | 018 | 0 | Epi/Stroma | 2545 |
| DSP-1001660010710-A-F03 | 019 | 0 | Immune | 95 |
| DSP-1001660010710-A-F04 | 019 | 0 | Epi/Stroma | 3041 |
| DSP-1001660010710-A-F05 | 020 | 0 | Immune | 181 |
| DSP-1001660010710-A-F06 | 020 | 0 | Epi/Stroma | 1962 |
| DSP-1001660010710-A-F07 | 021 | 0 | Immune | 144 |
| DSP-1001660010710-A-F08 | 021 | 0 | Epi/Stroma | 1573 |
| DSP-1001660010710-A-F09 | 022 | 0 | Immune | 444 |
| DSP-1001660010710-A-F10 | 022 | 0 | Epi/Stroma | 1505 |
| DSP-1001660010710-A-F11 | 023 | 0 | Immune | 338 |
| DSP-1001660010710-A-F12 | 023 | 0 | Epi/Stroma | 1684 |
| DSP-1001660010710-A-G01 | 024 | 0 | Immune | 617 |
| DSP-1001660010710-A-G02 | 024 | 0 | Epi/Stroma | 1263 |
| DSP-1001660010710-A-G03 | 025 | 0 | Immune | 941 |
| DSP-1001660010710-A-G04 | 025 | 0 | Epi/Stroma | 2321 |
| DSP-1001660010710-A-G05 | 026 | 0 | Immune | 237 |
| DSP-1001660010710-A-G06 | 026 | 0 | Epi/Stroma | 2353 |
| DSP-1001660010710-A-G07 | 027 | 0 | Immune | 216 |
| DSP-1001660010710-A-G08 | 027 | 0 | Epi/Stroma | 1782 |
| DSP-1001660010710-A-G09 | 028 | 0 | Immune | 481 |
| DSP-1001660010710-A-G10 | 028 | 0 | Epi/Stroma | 1621 |
| DSP-1001660010710-A-G11 | 029 | 1 | Immune | 926 |
| DSP-1001660010710-A-G12 | 029 | 1 | Epi/Stroma | 807 |
| DSP-1001660010710-A-H01 | 030 | 0 | Immune | 852 |
| DSP-1001660010710-A-H02 | 030 | 0 | Epi/Stroma | 2242 |
| DSP-1001660010710-A-H03 | 001 | 1 | Immune | 405 |
| DSP-1001660010710-A-H04 | 001 | 1 | Epi/Stroma | 669 |

| DSP-1001660010710-A-H05 | 002 | 1 | Immune | 583 |
| --- | --- | --- | --- | --- |
| DSP-1001660010710-A-H06 | 002 | 1 | Epi/Stroma | 2273 |
| DSP-1001660010710-A-H07 | 003 | 0 | Immune | 493 |
| DSP-1001660010710-A-H08 | 003 | 0 | Epi/Stroma | 1443 |
| DSP-1001660010710-A-H09 | 004 | 0 | Immune | 474 |
| DSP-1001660010710-A-H10 | 004 | 0 | Epi/Stroma | 1776 |
| DSP-1001660010711-B-A02 | 001 | 1 | Immune | 158 |
| DSP-1001660010711-B-A03 | 001 | 1 | Epi/Stroma | 631 |
| DSP-1001660010711-B-A04 | 002 | 1 | Immune | 187 |
| DSP-1001660010711-B-A05 | 002 | 1 | Epi/Stroma | 1765 |
| DSP-1001660010711-B-A06 | 003 | 1 | Immune | 268 |
| DSP-1001660010711-B-A07 | 003 | 1 | Epi/Stroma | 500 |
| DSP-1001660010711-B-A08 | 004 | 1 | Immune | 98 |
| DSP-1001660010711-B-A09 | 004 | 1 | Epi/Stroma | 1252 |
| DSP-1001660010711-B-A10 | 005 | 1 | Immune | 163 |
| DSP-1001660010711-B-A11 | 005 | 1 | Epi/Stroma | 914 |
| DSP-1001660010711-B-A12 | 006 | 0 | Immune | 210 |
| DSP-1001660010711-B-B01 | 006 | 0 | Epi/Stroma | 1076 |
| DSP-1001660010711-B-B02 | 007 | 0 | Immune | 289 |
| DSP-1001660010711-B-B03 | 007 | 0 | Epi/Stroma | 2046 |
| DSP-1001660010711-B-B04 | 008 | 0 | Immune | 361 |
| DSP-1001660010711-B-B05 | 008 | 0 | Epi/Stroma | 897 |
| DSP-1001660010711-B-B06 | 009 | 0 | Immune | 179 |
| DSP-1001660010711-B-B07 | 009 | 0 | Epi/Stroma | 1678 |
| DSP-1001660010711-B-B08 | 010 | 0 | Immune | 133 |
| DSP-1001660010711-B-B09 | 010 | 0 | Epi/Stroma | 1364 |
| DSP-1001660010711-B-B10 | 001 | 1 | Immune | 794 |
| DSP-1001660010711-B-B11 | 001 | 1 | Epi/Stroma | 3388 |
| DSP-1001660010711-B-B12 | 002 | 1 | Immune | 387 |
| DSP-1001660010711-B-C01 | 002 | 1 | Epi/Stroma | 2021 |
| DSP-1001660010711-B-C02 | 003 | 1 | Immune | 106 |
| DSP-1001660010711-B-C03 | 003 | 1 | Epi/Stroma | 205 |
| DSP-1001660010711-B-C04 | 004 | 1 | Immune | 547 |
| DSP-1001660010711-B-C05 | 004 | 1 | Epi/Stroma | 1784 |
| DSP-1001660010711-B-C06 | 005 | 1 | Immune | 710 |
| DSP-1001660010711-B-C07 | 005 | 1 | Epi/Stroma | 1682 |
| DSP-1001660010711-B-C08 | 006 | 1 | Immune | 330 |
| DSP-1001660010711-B-C09 | 006 | 1 | Epi/Stroma | 3197 |
| DSP-1001660010711-B-C10 | 007 | 1 | Immune | 74 |
| DSP-1001660010711-B-C11 | 007 | 1 | Epi/Stroma | 1578 |
| DSP-1001660010711-B-C12 | 008 | 1 | Immune | 224 |
| DSP-1001660010711-B-D01 | 008 | 1 | Epi/Stroma | 1435 |
| DSP-1001660010711-B-D02 | 009 | 0 | Immune | 107 |

| DSP-1001660010711-B-D03 | 009 | 0 | Epi/Stroma | 1552 |
| --- | --- | --- | --- | --- |
| DSP-1001660010711-B-D04 | 010 | 0 | Immune | 372 |
| DSP-1001660010711-B-D05 | 010 | 0 | Epi/Stroma | 1364 |
| DSP-1001660010711-B-D06 | 011 | 0 | Immune | 483 |
| DSP-1001660010711-B-D07 | 011 | 0 | Epi/Stroma | 2231 |
| DSP-1001660010711-B-D08 | 012 | 0 | Immune | 499 |
| DSP-1001660010711-B-D09 | 012 | 0 | Epi/Stroma | 1952 |
| DSP-1001660010711-B-D10 | 013 | 0 | Immune | 154 |
| DSP-1001660010711-B-D11 | 013 | 0 | Epi/Stroma | 1568 |
| DSP-1001660010711-B-D12 | 014 | 0 | Immune | 459 |
| DSP-1001660010711-B-E01 | 014 | 0 | Epi/Stroma | 2022 |
| DSP-1001660010711-B-E02 | 015 | 0 | Immune | 572 |
| DSP-1001660010711-B-E03 | 015 | 0 | Epi/Stroma | 2292 |
| DSP-1001660010711-B-E04 | 016 | 0 | Epi/Stroma | 1052 |
| DSP-1001660010711-B-E05 | 001 | 1 | Epi/Stroma | 2673 |
| DSP-1001660010711-B-E06 | 002 | 1 | Epi/Stroma | 2520 |
| DSP-1001660010711-B-E07 | 003 | 1 | Immune | 76 |
| DSP-1001660010711-B-E08 | 003 | 1 | Epi/Stroma | 1373 |
| DSP-1001660010711-B-E09 | 004 | 1 | Immune | 98 |
| DSP-1001660010711-B-E10 | 004 | 1 | Epi/Stroma | 1371 |
| DSP-1001660010711-B-E11 | 005 | 1 | Epi/Stroma | 1052 |
| DSP-1001660010711-B-E12 | 006 | 1 | Epi/Stroma | 1904 |
| DSP-1001660010711-B-F01 | 007 | 1 | Epi/Stroma | 822 |
| DSP-1001660010711-B-F02 | 008 | 1 | Epi/Stroma | 1465 |
| DSP-1001660010711-B-F03 | 009 | 1 | Epi/Stroma | 2610 |
| DSP-1001660010711-B-F04 | 010 | 1 | Immune | 90 |
| DSP-1001660010711-B-F05 | 010 | 1 | Epi/Stroma | 1064 |
| DSP-1001660010711-B-F06 | 011 | 1 | Immune | 61 |
| DSP-1001660010711-B-F07 | 011 | 1 | Epi/Stroma | 1258 |
| DSP-1001660010711-B-F08 | 012 | 1 | Immune | 318 |
| DSP-1001660010711-B-F09 | 012 | 1 | Epi/Stroma | 796 |
| DSP-1001660010711-B-F10 | 013 | 0 | Epi/Stroma | 1753 |
| DSP-1001660010711-B-F11 | 014 | 0 | Epi/Stroma | 1496 |
| DSP-1001660010711-B-F12 | 015 | 0 | Epi/Stroma | 765 |
| DSP-1001660010711-B-G01 | 016 | 0 | Epi/Stroma | 1057 |
| DSP-1001660010711-B-G02 | 017 | 0 | Epi/Stroma | 1905 |
| DSP-1001660010711-B-G03 | 018 | 0 | Immune | 596 |
| DSP-1001660010711-B-G04 | 018 | 0 | Epi/Stroma | 1875 |
| DSP-1001660010711-B-G05 | 019 | 0 | Epi/Stroma | 2854 |
| DSP-1001660010711-B-G06 | 020 | 0 | Epi/Stroma | 1096 |
| DSP-1001660010711-B-G07 | 021 | 0 | Epi/Stroma | 1272 |
| DSP-1001660010711-B-G08 | 022 | 0 | Immune | 3 |
| DSP-1001660010711-B-G09 | 022 | 0 | Epi/Stroma | 1097 |
| DSP-1001660010711-B-G10 | 023 | 0 | Immune | 9 |

| DSP-1001660010711-B-G11 | 023 | 0 | Epi/Stroma | 2107 |
| --- | --- | --- | --- | --- |
| DSP-1001660010711-B-G12 | 024 | 0 | Epi/Stroma | 1656 |
| DSP-1001660010711-B-H02 | 025 | 1 | Epi/Stroma | 1880 |
| DSP-1001660010711-B-H03 | 026 | 0 | Epi/Stroma | 1274 |
| DSP-1001660010711-B-H04 | 001 | 1 | Immune | 901 |
| DSP-1001660010711-B-H05 | 001 | 1 | Epi/Stroma | 639 |
| DSP-1001660010711-B-H06 | 002 | 1 | Immune | 147 |
| DSP-1001660010711-B-H07 | 002 | 1 | Epi/Stroma | 119 |
| DSP-1001660010711-B-H08 | 003 | 0 | Immune | 1000 |
| DSP-1001660010711-B-H09 | 003 | 0 | Epi/Stroma | 1302 |
| DSP-1001660010711-B-H10 | 004 | 0 | Immune | 1043 |
| DSP-1001660010711-B-H11 | 004 | 0 | Epi/Stroma | 2418 |

| **Fuso Score** | **Area** | **Reads (raw)** |
| --- | --- | --- |
| 0.834824969 | 44161.01 | 290065 |
| 0.834824969 | 259818.51 | 1101490 |
| 0.64338202 | 23807.85 | 5031776 |
| 0.64338202 | 215170.94 | 850450 |
| 0.642706645 | 16801.96 | 1537407 |
| 0.642706645 | 132320.3 | 6113922 |
| 0.506563574 | 24284.54 | 2878973 |
| 0.506563574 | 185538.07 | 3542504 |
| 0.073269417 | 96709.56 | 730040 |
| 0.073269417 | 96002.79 | 2350023 |
| 0.088005215 | 23511.38 | 554330 |
| 0.088005215 | 166090.03 | 1768762 |
| Artefact(3.43060969179 | 36539.67 | 1121452 |
| Artefact(3.43060969179 | 191687.57 | 4661332 |
| Artefact(0.70189744668 | 17263.92 | 700180 |
| Artefact(0.70189744668 | 216699.45 | 804203 |
| 2.975573911 | 151013.47 | 1315916 |
| 2.975573911 | 67300.95 | 422567 |
| 2.319110479 | 49278.61 | 216593 |
| 0.62785905 | 97234.18 | 4202337 |
| 2.815791383 | 109893.46 | 2767208 |
| 0.099471503 | 127636.81 | 4410996 |
| 0.031861779 | 120004.78 | 1054284 |
| 0.155948399 | 191775.49 | 3637374 |
| 0.068728899 | 180088.21 | 2189310 |
| 30.55925601 | 54429.07 | 359787 |
| 30.55925601 | 128036.59 | 606186 |
| 24.72363283 | 30925.95 | 354597 |
| 24.72363283 | 158739.42 | 11222952 |
| 24.12240848 | 93168.41 | 1559804 |
| 24.12240848 | 148568.5 | 1557639 |
| 9.855942328 | 49066.33 | 3514602 |
| 9.855942328 | 113456.8 | 555605 |
| 8.538376119 | 81471.09 | 222528 |
| 8.538376119 | 170183.01 | 278877 |
| 3.264005536 | 30635.79 | 2969130 |
| 3.264005536 | 167095.06 | 370689 |
| 2.133347345 | 53932.15 | 4525106 |
| 2.133347345 | 137676.25 | 5036837 |
| 2.93907615 | 43615.99 | 695949 |
| 2.93907615 | 150722.02 | 239343 |
| 13.13226759 | 30519.86 | 1746257 |

| 13.13226759 | 133298.45 | 2642390 |
| --- | --- | --- |
| 1.200350205 | 8257.36 | 1999029 |
| 1.200350205 | 172681.74 | 2359814 |
| 6.915241776 | 62753.31 | 1860827 |
| 6.915241776 | 88576.56 | 703608 |
| 4.131053928 | 20356.4 | 3575724 |
| 4.131053928 | 66335.91 | 1121573 |
| 0.889360454 | 59771.9 | 2894889 |
| 0.889360454 | 119854.68 | 3947529 |
| 0.5244411414568264 | 24955.85 | 937441 |
| 0.5244411414568264 | 116657.6 | 806626 |
| 0.430118537 | 30630.94 | 3426887 |
| 0.430118537 | 204174.56 | 5451975 |
| 0.414715624 | 20760.55 | 811425 |
| 0.414715624 | 181883.4 | 658497 |
| 1.237421875 | 23946.13 | 587550 |
| 1.237421875 | 137854.68 | 461195 |
| 1.397360532 | 22157.58 | 3198579 |
| 1.397360532 | 180241.71 | 3387872 |
| 0.65244396 | 23567.73 | 395230 |
| 0.65244396 | 156356.79 | 5118890 |
| 0.559680742 | 19320.93 | 288815 |
| 0.559680742 | 134614.04 | 2492813 |
| 0.507157392 | 65332.34 | 1453187 |
| 0.507157392 | 88874.49 | 4124586 |
| 0.168323859 | 31205.91 | 867781 |
| 0.168323859 | 169655.48 | 3265728 |
| 0.239438198 | 88170.63 | 504361 |
| 0.239438198 | 96460.05 | 320533 |
| 0.728884845 | 82470.93 | 860182 |
| 0.728884845 | 138309.35 | 2875865 |
| 2.44789253 | 40444.33 | 1397262 |
| 2.44789253 | 139145.01 | 206924 |
| 2.07578506 | 19993.7 | 3348792 |
| 2.07578506 | 147111.56 | 425484 |
| 3.332060169 | 78639.46 | 2628264 |
| 3.332060169 | 116350.77 | 1223587 |
| 5.269538417 | 117984.2 | 265996 |
| 5.269538417 | 75548.76 | 719180 |
| 2.462157596 | 97996.98 | 3753678 |
| 2.462157596 | 117139.8 | 835452 |
| 1.97060644 | 75221.36 | 5528977 |
| 1.97060644 | 67253.35 | 159204 |

| 1.054655704 | 68559.38 | 1525053 |
| --- | --- | --- |
| 1.054655704 | 173049.45 | 446766 |
| 0.340757724 | 43605.95 | 2728142 |
| 0.340757724 | 124415.93 | 1162070 |
| 0.086245304 | 42484.34 | 2540958 |
| 0.086245304 | 127958.54 | 1744814 |
| 1.078737153 | 25698.57 | 366787 |
| 1.078737153 | 104548.69 | 298933 |
| 0.536203225 | 18832.91 | 1706117 |
| 0.536203225 | 229337.51 | 435203 |
| 0.605193951 | 45964.62 | 740178 |
| 0.605193951 | 154708.78 | 230591 |
| 1.136271314 | 32011.45 | 3019054 |
| 1.136271314 | 240896.88 | 4320616 |
| 2.731018752 | 59271.9 | 1468836 |
| 2.731018752 | 239789.19 | 3926965 |
| 0.081778202 | 20354.78 | 344061 |
| 0.081778202 | 163737.84 | 728877 |
| 0.04100857 | 23206.81 | 441786 |
| 0.04100857 | 215697.01 | 2628072 |
| 0.01745658 | 32876.75 | 160882 |
| 0.01745658 | 190138.98 | 3072167 |
| 0.0244859 | 20702.74 | 994898 |
| 0.0244859 | 251165.59 | 477463 |
| 0.060510934 | 84464.8 | 1387711 |
| 0.060510934 | 150166.96 | 2055282 |
| 15.38114858 | 106416.42 | 4556000 |
| 15.38114858 | 240139.1 | 809905 |
| 17.64750926 | 62079.56 | 1014164 |
| 17.64750926 | 167516.37 | 1650234 |
| 2.363669348 | 52268.11 | 688759 |
| 2.363669348 | 113348.15 | 1152914 |
| 30.44685398 | 149615.96 | 1629494 |
| 30.44685398 | 147376.46 | 5770943 |
| 10.65936588 | 70109.1 | 4149276 |
| 10.65936588 | 198593.56 | 27608 |
| 7.641250229 | 68401.02 | 2908498 |
| 7.641250229 | 270490.4 | 5035301 |
| 37.95031427 | 93175.37 | 2813278 |
| 37.95031427 | 229664.91 | 3409369 |
| 14.42223433 | 140194.89 | 2560963 |
| 14.42223433 | 140373.32 | 1151636 |
| 0.166105145 | 69445.56 | 2099841 |

| 0.166105145 | 173707 | 122979 |
| --- | --- | --- |
| 1.153207454 | 98732.74 | 2336261 |
| 1.153207454 | 110810.24 | 1137140 |
| 0.089941473 | 44732.91 | 733118 |
| 0.089941473 | 257547.93 | 3141530 |
| 0.017020108 | 58901.26 | 1930950 |
| 0.017020108 | 232827.51 | 1193675 |
| 2.187915487 | 31095.97 | 3256496 |
| 2.187915487 | 186373.4 | 6267100 |
| 0.240566695 | 70013.73 | 437752 |
| 0.240566695 | 188297.32 | 1485707 |
| 0.50293764 | 48023.74 | 1012199 |
| 0.50293764 | 216408.32 | 3739902 |
| 0.00694995 | 171544.1 | 5287251 |
| 5.893385317 | 237089.04 | 3456660 |
| 4.51744824 | 199616.88 | 449576 |
| 1.270436746 | 28904.89 | 3508254 |
| 1.270436746 | 83777.13 | 264096 |
| 4.722952162 | 40122.92 | 552306 |
| 4.722952162 | 88891.98 | 5956227 |
| 1.244654231 | 87108.77 | 3977822 |
| 0.66277555 | 169798.29 | 224008 |
| 0.992328932 | 90291.27 | 1050810 |
| 0.453992944 | 147071.08 | 3800222 |
| 0.3225564374442689 | 298895.28 | 2098732 |
| 1.823943255 | 20695.62 | 2765769 |
| 1.823943255 | 89839.52 | 1937448 |
| 0.905538546 | 14446.86 | 1406381 |
| 0.905538546 | 90037.55 | 2935033 |
| 0.801655256 | 44845.6 | 2627931 |
| 0.801655256 | 31278.29 | 3332536 |
| 0.017962908 | 166380.35 | 1925654 |
| 0 | 90442.34 | 720154 |
| 0.004150508 | 47881.74 | 618886 |
| 0.002958112 | 63889.33 | 1278267 |
| 3.55E-05 | 144516.82 | 2763162 |
| 3.97E-05 | 42479.49 | 1703975 |
| 3.97E-05 | 132900.78 | 835919 |
| 4.09E-05 | 254907.04 | 3382169 |
| 0.001365416 | 88337.73 | 2378200 |
| 0.00061485 | 61222.69 | 5995741 |
| 0.013151991 | 7377.82 | 4303803 |
| 0.013151991 | 64765.79 | 5100444 |
| 0.18501298 | 18990.45 | 374341 |

| 0.18501298 | 178555.17 | 1393992 |
| --- | --- | --- |
| 0.011288247 | 210310.47 | 999836 |
| 0.42964335611954785 | 228994.57 | 941876 |
| 9.419215859698896e-05 | 134766.25 | 3129934 |
| 0.913928061 | 187368.56 | 247074 |
| 0.913928061 | 25955.22 | 1982322 |
| 1.016720089 | 112207.6 | 6008255 |
| 1.016720089 | 14378.37 | 4658265 |
| 0.004083841 | 111735.6 | 596309 |
| 0.004083841 | 35001.44 | 378933 |
| 0.754714284 | 122560.02 | 1743466 |
| 0.754714284 | 102843.2 | 1228531 |
